# Social epidemiology of sports and extracurricular activities in early adolescents

**DOI:** 10.1038/s41390-025-04099-6

**Published:** 2025-05-04

**Authors:** Jason M. Nagata, Jennifer H. Wong, Christiane K. Helmer, Thang Diep, Sydnie K. Domingue, Abubakr A. A. Al-shoaibi, Kyle T. Ganson, Alexander Testa, Erin E. Dooley, Holly C. Gooding, Fiona C. Baker, Kelley Pettee Gabriel

**Affiliations:** 1 Department of Pediatrics, University of California, San Francisco, CA USA; 2 Factor-Inwentash Faculty of Social Work, University of Toronto, Toronto, Toronto, ON Canada; 3 Department of Management, Policy and Community Health, University of Texas Health Science Center at Houston, Houston, TX USA; 4 Department of Epidemiology, University of Alabama at Birmingham, Birmingham, AL USA; 5 Division of General Pediatrics and Adolescent Medicine, Department of Pediatrics, Emory University School of Medicine, Atlanta, GA USA; 6 Center for Health Sciences, SRI International, Menlo Park, CA USA

## Abstract

**Background:**

This study examines the social epidemiology of sports and extracurricular activities in early adolescents (9–14 years) using a diverse national U.S. sample.

**Methods:**

We examined data from baseline (2016–2018, ages 9–10) to Year 3 follow-up (2019–2021) of the Adolescent Brain and Cognitive Development (ABCD) Study (*N* = 11,270). Multivariable linear regression models with standardized betas estimated adjusted cross-sectional associations between sociodemographic factors (age, biological sex, sexual orientation, race and ethnicity, household income, parental education) and physical activity (metabolic equivalent of task (MET)-hours(h)/week) through sports and extracurricular activities at baseline and Year 3.

**Results:**

Average MET-h/week decreased from 15.5 (±18.7) in Year 1 follow-up to 13.0 (±19.1) in Year 3 follow-up. Female sex, gay/bisexual sexual orientation, lower household income, and lower parental education were associated with lower MET-h/week at baseline. Male adolescents were more likely to play soccer and baseball/softball; female adolescents were more likely to play a musical instrument and participate in swimming/water polo and ballet/dance. Among the most common sports and extracurricular activities, lower parental income and education were associated with lower participation.

**Conclusion:**

This study of early adolescents identified sociodemographic differences in sports and extracurricular activities that can inform targeted interventions to reduce these disparities.

**Impact:**

- This study examines the trends and social epidemiology of sports and extracurricular activities in early adolescents using a demographically diverse national U.S. sample.
- Average early adolescent physical activity decreased across three years of follow-up.
- Soccer, musical instruments, swimming/water polo, baseball/softball, and ballet/dance were the most common activities at baseline.
- Female sex, gay/bisexual sexual orientation, lower household income, and lower parental education were associated with lower weekly physical activity at baseline.

## Introduction

Adolescence is a crucial developmental stage for establishing physical activity habits that can persist into adulthood,^1–3^ which can then improve health outcomes and quality of life. Adolescents who engage in sufficient levels of physical activity are more likely to have higher academic performance^4^ and are less likely to engage in behaviors such as drug and alcohol use, smoking, and drunk driving.^5^ Furthermore, engagement in physical activity reduces the risk of cardiovascular disease, stroke, type 2 diabetes,^6^ depressive symptoms, suicide attempts, and premature death later in life.^2,7,8^ Notably, one study found that physical activity during youth was a stronger predictor of lower incidence of arterial hypertension and type 2 diabetes in adulthood, compared to physical activity in adulthood.^4,5,9^ Additionally, sports participation, a common form of physical activity among youth, has been linked with improved cardiovascular risk markers in adulthood, including lower carotid intima-media index and better cardiac autonomic modulation, regardless of current adult physical activity levels.^10,11^ Likewise, adults who engaged in sports during early life had lower body fat and higher current physical activity levels compared to those who did not.^12^ These findings highlight the lasting impact of physical activity and sports participation during these formative years, reinforcing the importance of encouraging and supporting active lifestyles from an early age. Accordingly, the American Academy of Pediatrics also recommends sports engagement as an avenue to meet physical activity guidelines.^13^

Current guidelines by the United States Department of Health and Human Services and the World Health Organization recommend at least 60 min of moderate-to-vigorous intensity physical activity (MVPA) daily for children and adolescents ages 5–17 years.^14,15^ Self-reported data from US high school students reveal that more than 70% of adolescents do not meet these recommendations.^16^ Global trends also show that 81% of adolescents are insufficiently active.^17^ During the COVID-19 pandemic, evidence from cohort studies documented an even lower prevalence of physical activity.^18^ Specifically, in the Adolescent Brain and Cognitive Development (ABCD) Study, less than 5% of early adolescent participants met physical activity guidelines in the later stage of the pandemic.^19^ The COVID-19 pandemic further exacerbated existing disparities in early adolescent physical activity as a result of the closure of facilities and organized sports and lack of access to school-based physical education, with age, sex, and socioeconomic status as key determinants of physical activity in children and adolescents.^18^

Social epidemiology can provide insights into the impact of structural and sociodemographic factors on health behaviors and outcomes with the goal of understanding health disparities.^20–22^ Prior research has shown that sociodemographic characteristics are important predictors of physical activity in adolescents, with data highlighting that racial and ethnic minority,^17,23,24^ female,^17,23,24^ and sexual minority adolescents^25,26^ are less likely to be physically active. Participation in team sports is also less common among sexual minority and female high school students.^27^ Additionally, children and adolescents from low-affluence families are less likely to meet physical activity recommendations and participate in sports compared to those from high-affluence families.^28,29^

Although prior research has established a foundation for understanding disparities in physical activity among adolescents, gaps remain in the literature regarding the social epidemiology of sports participation, the primary form of physical exercise in early adolescence. For instance, previous studies mostly relied on self-reported questionnaires asking youth to recall their overall physical activity levels,^23,24,26–28,30,31^ with few examining the specific categories such as different types of sports and extracurricular activities.^13,24,27,32^ Thus, determining the prevalence and sociodemographic associations of specific sports and extracurricular activities can further guide the implementation of targeted physical activity and sports interventions for early adolescents. To the best of our knowledge, no studies have evaluated the social epidemiology of participation in different sports and extracurricular activities, which is a comprehensive measure that may provide more accurate recall. Additionally, no studies have investigated engagement in sports and extracurricular activities before and during the pandemic in the same cohort of early adolescents.

Given the numerous lifetime health benefits of sports participation during adolescence, a thorough evaluation of the most commonly endorsed sports and extracurricular activities and upstream sociodemographic factors associated with endorsement of these activities among early adolescents is warranted. This is an important public health priority given that physical activity levels in adolescents often decline as they transition from middle school to high school and beyond.^16,23^ In the present study, we aim to address this priority by examining the trends and social epidemiology of sports and extracurricular activities in early adolescents using a demographically diverse national US cohort.

## Methods

### Study population

We examined data from the ABCD Study, the largest longitudinal study of adolescent brain health and development in the US, from baseline (2016–2018) through Year 3 follow-up (2019–2021). Across 21 demographically diverse sites from four U.S. regions, 11,875 children aged 9–10 years were recruited at baseline. Out of this sample, we excluded 194 participants missing sports and extracurricular activities data and 498 participants missing sociodemographic characteristics from baseline or Year 3 follow-up, resulting in a sample size of 11,270 participants (Appendix A). The University of California, San Diego, and each local study site granted Institutional Review Board (IRB) approval. Written informed consent was provided by parents/caregivers, and written assent was provided by children.

### Sociodemographic variables

We analyzed sociodemographic variables collected at baseline and Year 3 follow-up for our cross-sectional analyses, which were conducted separately for each time point. Age of participant, sex assigned at birth (female or male), sexual orientation (heterosexual, maybe gay/bisexual, gay/bisexual, don’t understand the question, or decline to answer), race and ethnicity (Asian, Black, Latino/Hispanic, Native American, White, other), household income (six categories), and parental education status (high school education or less vs. college education or more) were self-reported by the participants and/or their caregivers. For both baseline and Year 3 follow-up, sexual orientation was analyzed using data at Year 3 follow-up only, since it may be more reflective of the adolescents’ true sexual orientation as they may have greater awareness of their sexual identity. Because of partial disruptions in data collection during the COVID-19 pandemic, we used the last observation carried forward method to impute missing data for sexual orientation, household income, and parental education at Year 3 follow-up.

### Outcome

The Sports Activity Involvement Questionnaire (SAI-Q), based on the Vermont Health and Behavioral Questionnaire (VHBQ) and the Dutch Health Behavioral Questionnaire (DHBQ),^33^ assesses lifetime (baseline) and past year (since last visit) involvement in 23 different sports, extracurricular activities like music, and other hobbies like drawing. The SAI-Q was administered to parents/caregivers at baseline and each follow-up year. At baseline, they were asked whether the child had participated in each activity in their lifetime, and at each follow-up year, they were asked if their child had participated since last visit (yes/no). For each activity, they were asked to report (1) time spent (minutes) per session (duration per session), (2) number of days per week participating (days per week), (3) number of months per year (months per year), and (4) number of years participated (at baseline). We assigned an age-specific metabolic equivalent of task (MET) value to each sport and extracurricular activity based on the 2011 Youth Compendium of Physical Activities to quantify the energy costs of physical activities as multiples of resting metabolic rate.^34,35^ The MET values ranged from 0 to 10, with higher METs corresponding to more vigorous intensity physical activity. For each sport and extracurricular activity endorsed, the mean hours per week of participation was calculated as: [(duration per session * days per week * months per year*4.33)/(52*60)] where 4.33 is the number of weeks in each month, 52 is the number of weeks in a year, and 60 is the number of minutes per hour. The mean hours per week of participation were multiplied by the assigned MET value to calculate an individual MET-h/week score for each sport and extracurricular activity. The activity-specific MET-h/week scores were summed across each endorsed activity type to derive an estimate of overall physical activity (also expressed as MET-h/week).

The U.S. Department of Health and Human Services advises adolescents to engage in 60 min or more of MVPA per day.^14^ In our study, we calculated a cutoff value for meeting physical activity guidelines by incorporating the lower limit of moderate-intensity activity, 3.0 METs,^14^ because it is the absolute minimum for meeting physical activity guidelines. We multiplied 3.0 METs by 420 min per week (based on the recommended 60 min of exercise per day [*7]), resulting in 1260 MET-min/week. We divided this value by 60 min to get our final cutoff value of 21 MET-h/week. Participants met physical activity guidelines if they had ≥21 MET-h/week after excluding activities with METs below 3.0 (lower limit of moderate-intensity activity), including playing a musical instrument and drawing, from their total MET-h/week score.^14,34,35^

### Statistical analyses

Data analyses were conducted using Stata 18 (StataCorp, College Station, TX) with data from baseline (2016–2018) and the first three follow-up years: Year 1 follow-up (2017–2019), Year 2 follow-up (2018–2020), and Year 3 follow-up (2019–2021). Given a change in the way questions were asked about activity involvement from baseline (lifetime) to follow-up years (since last visit), longitudinal analyses were only performed on data from Year 1 to Year 3. In addition, cross-sectional analyses were performed separately for both baseline and Year 3. Descriptive statistics were calculated by measuring the mean, standard deviation, and percentages of sociodemographic factors, participation in each sport and extracurricular activity, and MET-h/week sum scores. The prevalence of each sport and extracurricular activity was also stratified by sex and race. Time trend analyses using chi-square tests and linear regression were conducted to determine if MET-h/week and sport and extracurricular activity participation differed across Year 1 follow-up to Year 3 follow-up. Multivariable linear regression models with standardized beta coefficients were conducted to estimate the cross-sectional associations between sociodemographic factors (age, sex, sexual orientation, race and ethnicity, household income, parental education) and continuous MET-h/week sum score separately for baseline (Model 1) and Year 3 follow-up (Model 2). Multivariable linear regression models with standardized beta coefficients were also conducted to estimate the cross-sectional associations between sociodemographic factors and participation in the five most common sports and extracurricular activities at baseline. To address potential confounding, models were adjusted for sociodemographic factors for respective years and study sites. Predetermined ABCD sampling weights based on the American Community Survey from the US Census were utilized in the analysis to represent population estimates. Statistical significance was based on a two-sided *p* < 0.05.

## Results

Of the total sample size included (*N* = 11,270), at baseline, the mean age was 9.9 (±0.6) years, 48.8% of the participants were female, 46.4% were racial and ethnic minorities, and 56.2% came from a household with an annual income of $75,000 or less (Table 1). The average MET-h/week sum score significantly declined over time: 15.5 (±18.7) at Year 1 follow-up, 15.1 (±19.4) at Year 2 follow-up, and 13.0 (±19.1) at Year 3 follow-up (Table 1). The prevalence of participants meeting physical activity guidelines significantly declined across the three follow-up years: 22.0% at Year 1 follow-up, 21.8% at Year 2 follow-up, and 18.3% at Year 3 follow-up (Table 1).

**Table 1:** Sociodemographic characteristics of 11,270 Adolescent Brain Cognitive Development (ABCD) Study participants at baseline (2016–2018) or at follow-up years, where indicated^a^

Sociodemographic characteristics	Mean (SD) / % (95% CI)	*p*^b^
Age (years)	9.9 (0.6)	
Biological sex at birth		
Female	48.8% (47.7%, 49.8%)	
Male	51.2% (50.2%, 52.3%)	
Sexual orientation (Year 3 follow-up)		
Heterosexual	82.5% (81.7%, 83.2%)	
Maybe gay/bisexual	5.2% (4.7%, 5.6%)	
Gay/bisexual	8.3% (7.7%, 8.9%)	
Don’t understand the question	2.5% (2.2%, 2.8%)	
Refuse to answer	1.6% (1.4%, 1.9%)	
Race and ethnicity		
Asian	5.5% (5.0%, 6.0%)	
Black	16.6% (15.9%, 17.4%)	
Latino/Hispanic	19.7% (18.8%, 20.5%)	
Native American	3.2% (2.8%, 3.6%)	
Other	1.4% (1.2%, 1.8%)	
White	53.6% (52.6%, 54.7%)	
Household income		
$24,999 or less	18.3% (17.5%, 19.2%)	
$25,000 to $49,999	20.3% (19.4%, 21.3%)	
$50,000 to $74,999	17.6% (16.7%, 18.4%)	
$75,000 to $99,999	13.5% (12.9%, 14.2%)	
$100,000 to $199,999	22.8% (22.0%, 23.6%)	
$200,000 or greater	7.5% (7.0%, 7.9%)	
Parent’s highest education		
High school education or less	18.9% (18.0%, 19.7%)	
College education or more	81.1% (80.3%, 82.0%)	
MET-h/week sum score^c^		<0.001
Baseline	19.3 (21.6)	
Year 1 follow-up	15.5 (18.7)	
Year 2 follow-up	15.1 (19.4)	
Year 3 follow-up	13.0 (19.1)	
Meeting physical activity guidelines^d^		<0.001
Baseline	28.5% (27.6%, 29.5%)	
Year 1 follow-up	22.0% (21.2%, 22.9%)	
Year 2 follow-up	21.8% (20.9%, 22.6%)	
Year 3 follow-up	18.3% (17.5%, 19.2%)	

*SD* standard deviation.

^a^Sampling weights were applied to yield representative estimates based on the American Community Survey from the US Census.

^b^P-values were calculated from a chi-square test and linear regression to determine whether MET-h/week and meeting physical activity guidelines significantly differed across Year 1 follow-up to Year 3 follow-up

^c^MET-h/week scores at baseline and follow-up years were calculated from lifetime and past year activity involvement, respectively

^d^Guidelines were met if the participant had ≥21 MET-h/week, the equivalent of ≥60 minutes of moderate-intensity physical activity per day

Table 2 shows the prevalence of participation in each sport and extracurricular activity from baseline to Year 3 follow-up. At baseline, the most common activities included soccer (38.3%), musical instrument (37.1%), swimming/water polo (30.5%), baseball/softball (25.9%), and ballet/dance (24.3%). At the Year 3 follow-up, the most common response was that participants had not engaged in any activity since their last visit (27.5%). Meanwhile, drawing (14.2%) and basketball (13.5%) emerged as some of the most common activities at Year 3 follow-up, replacing swimming/water polo and baseball/softball. Overall, participation in sports and extracurricular activities decreased from Year 1 follow-up to Year 3 follow-up. Appendix B shows the prevalence of participation in each sport and extracurricular activity stratified by sex, and Appendix C shows the prevalence stratified by race and ethnicity.

**Table 2:** Prevalence and 95% confidence intervals of participation in each sport and extracurricular activity in the Adolescent Brain Cognitive Development (ABCD) Study from baseline (lifetime participation) to Year 1, Year 2, and Year 3 follow-up (participation since last visit)

Activity	Baseline (lifetime)	Year 1 (since last visit)	Year 2 (since last visit)	Year 3 (since last visit)	*p*
Ballet, dance	24.3% (23.5%, 25.2%)	10.7% (10.1%, 11.4%)	8.3% (7.8%, 8.9%)	6.4% (5.9%, 7.0%)	<0.001
Baseball, softball	25.9% (25.0%, 26.8%)	13.5% (12.8%, 14.3%)	10.9% (10.3%, 11.6%)	8.4% (7.8%, 9.0%)	<0.001
Basketball	24.2% (23.3%, 25.0%)	19% (18.2%, 19.8%)	16.9% (16.1%, 17.7%)	13.5% (12.8%, 14.3%)	<0.001
Climbing	3.2% (2.8%, 3.5%)	2.6% (2.3%, 2.9%)	1.7% (1.5%, 2.0%)	1.1% (0.9%, 1.4%)	<0.001
Field hockey	0.6% (0.4%, 0.7%)	0.6% (0.5%, 0.8%)	0.7% (0.5%, 0.8%)	0.7% (0.5%, 0.8%)	0.896
Football	10.8% (10.2%, 11.5%)	8.2% (7.2%, 8.4%)	7.1% (6.6%, 7.7%)	5.7% (5.2%, 6.2%)	<0.001
Gymnastics	21.1% (20.3%, 22.0%)	7.8% (7.2%, 8.4%)	4.8% (4.4%, 5.3%)	3% (2.7%, 3.4%)	<0.001
Ice hockey	2.3% (2.0%, 2.6%)	1.5% (1.2%, 1.7%)	1.4% (1.2%, 1.7%)	1.4% (1.2%, 1.6%)	0.900
Horseback riding, polo	3.8% (3.4%, 4.2%)	2% (1.7%, 2.3%)	1.6% (1.3%, 1.8%)	1.4% (1.2%, 1.7%)	0.016
Ice or inline skating	5.8% (5.3%, 6.3%)	2.9% (2.6%, 3.3%)	1.5% (1.3%, 1.8%)	1.2% (0.9%, 1.4%)	<0.001
Martial arts	17.1% (16.3%, 17.9%)	6.7% (6.2%, 7.2%)	4.8% (4.4%, 5.3%)	3.8% (3.4%, 4.2%)	<0.001
Lacrosse	2.3% (2.1%, 2.6%)	2.2% (1.9%, 2.4%)	2% (1.8%, 2.3%)	1.5% (1.3%, 1.8%)	0.003
Rugby	0.3% (0.2%, 0.4%)	0.2% (0.2%, 0.4%)	0.1% (0.0%, 0.2%)	0.2% (0.1%, 0.3%)	0.119
Skateboarding	4% (3.6%, 4.5%)	2.9% (2.6%, 3.3%)	3.6% (3.2%, 4.0%)	4.2% (3.8%, 4.7%)	0.001
Skiing, snowboarding	6.2% (5.8%, 6.6%)	4.9% (4.5%, 5.3%)	4.7% (4.3%, 5.2%)	4.6% (4.2%, 5.0%)	0.474
Soccer	38.3% (37.3%, 39.3%)	21.6% (20.7%, 22.4%)	16.8% (16.1%, 17.6%)	13% (12.3%, 13.8%)	<0.001
Surfing	0.4% (0.3%, 0.6%)	0.4% (0.3%, 0.5%)	0.3% (0.2%, 0.4%)	0.4% (0.3%, 0.6%)	0.154
Swimming, water polo	30.5% (29.6%, 31.4%)	18.9% (18.1%, 19.8%)	12.2% (11.5%, 12.9%)	8.4% (7.8%, 9.0%)	<0.001
Tennis	6% (5.5%, 6.4%)	3.5% (3.1%, 3.9%)	3.3% (2.9%, 3.6%)	3% (2.7%, 3.4%)	0.254
Track, running, cross-country	6.3% (5.8%, 6.7%)	6.6% (6.1%, 7.1%)	6.4% (5.9%, 7.0%)	6% (5.5%, 6.6%)	0.314
Wrestling, mixed martial arts	2.9% (2.5%, 3.2%)	1.6% (1.4%, 1.9%)	1.5% (1.3%, 1.8%)	1.5% (1.2%, 1.8%)	0.705
Volleyball	2.6% (2.3%, 2.9%)	3.5% (3.2%, 3.9%)	4.6% (4.2%, 5.1%)	4.9% (4.5%, 5.4%)	<0.001
Yoga, tai chi	2.3% (2.0%, 2.6%)	1.2% (1.0%, 1.5%)	0.8% (0.6%, 1.0%)	0.8% (0.6%, 1.0%)	0.002
Musical instrument (singing, choir, guitar, piano, drums, violin, flute, band, rock band, orchestra)	37.1% (36.1%, 38.1%)	37.3% (36.3%, 38.4%)	35.4% (34.4%, 36.4%)	29.5% (28.5%, 30.5%)	<0.001
Drawing, painting, graphic art, photography, pottery, sculpting	20.3% (19.5%, 21.2%)	19.2% (18.4%, 20.1%)	15.5% (14.7%, 16.3%)	14.2% (13.5%, 15.0%)	<0.001
Drama, theater, acting, film	10.3% (9.7%, 10.9%)	9.1% (8.5%, 9.7%)	8.7% (8.1%, 9.3%)	6.6% (6.0%, 7.1%)	<0.001
Crafts like knitting, building model cars of airplanes	8.6% (8.0%, 9.2%)	7.5% (7.0%, 8.1%)	5.1% (4.7%, 5.6%)	4.4% (3.9%, 4.9%)	<0.001
Competitive games like chess, cards, or darts	10.3% (9.7%, 10.9%)	8.3% (7.7%, 8.9%)	5.4% (4.9%, 5.9%)	4.4% (3.9%, 4.9%)	<0.001
Hobbies like collecting stamps or coins	6.9% (6.4%, 7.4%)	4.6% (4.1%, 5.0%)	3.1% (2.7%, 3.5%)	2.3% (2.0%, 2.6%)	<0.001
My child has not participated in any of the above activities	12.3% (11.6%, 13.0%)	17% (16.2%, 17.9%)	21.4% (20.5%, 22.3%)	27.5% (26.5%, 28.5%)	<0.001

Sampling weights were applied to yield representative estimates based on the American Community Survey from the US Census. Baseline and follow-up years measure lifetime and past-year activity involvement, respectively. *P* values were calculated from chi-square tests to determine whether sports activity participation significantly differed from Year 1 follow-up to Year 3 follow-up.

Table 3 examines the relationship between sociodemographic factors and MET-h/week separately, at baseline and Year 3 follow-up, reported as standardized beta coefficients. At both assessments, older age was associated with higher MET-h/week. Compared to female sex, male sex was associated with higher MET-h/week at baseline (*β* = 0.11, 95% confidence interval [CI] 0.07, 0.15; *p* < 0.001). Sex was not significantly associated with MET-h/week at Year 3 follow-up. Compared to heterosexual participants, gay/bisexual sexual orientation was associated with lower MET-h/week at baseline and Year 3 follow-up. Compared to White race, at baseline, Asian race was associated with lower MET-h/week (*β* = −0.13, 95% CI −0.22, −0.04; *p* = 0.004), but there was no longer a difference at Year 3 follow-up. At Year 3 follow-up, Latino/Hispanic (*β* = –0.10, 95% CI −0.17, −0.03; *p* = 0.005) and Native American (*β* = −0.12, 95% CI −0.22, −0.01; *p* = 0.028) race and ethnicity were associated with lower MET-h/week, compared to White race. At baseline and Year 3 follow-up, household income of $24,999 or less, $25,000 to $49,999, $50,000 to $74,999, and $100,000 to $199,999 were associated with lower MET-h/week compared to household income of $200,000 or greater. At both baseline and Year 3 follow-up, compared to parental education of college education or higher, high school education or less was associated with lower MET-h/week. The sociodemographic associations for the five most common sports and extracurricular activities are shown in Appendix D. Among the most common sports and extracurricular activities (soccer, musical instrument, swimming/water polo, baseball/softball, and ballet/dance), male adolescents were more likely to play soccer and baseball/softball, and female adolescents were more likely to play a musical instrument and participate in swimming/water polo and ballet/dance. Participation in each of these activities varied by race and ethnicity. Lower parental income and education were associated with lower participation in these activities.

**Table 3:** Sociodemographic associations with MET-h/week in the Adolescent Brain Cognitive Development (ABCD) Study at baseline and Year 3 follow-up

	MET-h/week at baseline (Model 1)		MET-h/week at Year 3 (Model 2)	
Sociodemographic characteristics	Adjusted standardized β (95% CI)	*p*	Adjusted standardized β (95% CI)	*p*
Age	**0.11 (0.09, 0.13)**	**<0.001**	**0.08 (0.06, 0.10)**	**<0.001**
Biological sex at birth				
Female	reference		reference	
Male	**0.11 (0.07, 0.15)**	**<0.001**	−0.01 (−0.06, 0.03)	0.565
Sexual orientation (Year 3)				
Heterosexual	reference		reference	
Maybe gay/bisexual	−0.08 (−0.16, 0.01)	0.071	**−0.24 (−0.31, −0.17)**	**<0.001**
Gay/bisexual	**−0.10 (−0.17, −0.04)**	**0.003**	**−0.22 (−0.29, −0.16)**	**<0.001**
Don’t understand the question	−0.04 (−0.17, 0.08)	0.494	−0.11 (−0.23, 0.01)	0.063
Refuse to answer	−0.13 (−0.26, 0.00)	0.052	**−0.18 (−0.30, −0.06)**	**0.003**
Race and ethnicity				
Asian	**−0.13 (−0.22, −0.04)**	**0.004**	−0.03 (−0.16, 0.09)	0.627
Black	0.05 (−0.02, 0.12)	0.182	−0.06 (−0.13, 0.01)	0.082
Latino/Hispanic	−0.05 (−0.12, 0.02)	0.204	**−0.10 (−0.17, −0.03)**	**0.005**
Native American	−0.05 (−0.15, 0.05)	0.321	**−0.12 (−0.22, −0.01)**	**0.028**
Other	0.10 (−0.23, 0.42)	0.553	0.20 (−0.22, 0.63)	0.352
White	reference		reference	
Household income				
$24,999 or less	**−0.77 (−0.87, −0.68)**	**<0.001**	**−0.56 (−0.67, −0.44)**	**<0.001**
$25,000 to $49,999	**−0.70 (−0.79, −0.62)**	**<0.001**	**−0.55 (−0.65, −0.44)**	**<0.001**
$50,000 to $74,999	**−0.62 (−0.70, −0.54)**	**<0.001**	**−0.49 (−0.59, −0.39)**	**<0.001**
$75,000 to $99,999	**−0.50 (−0.58, −0.42)**	**<0.001**	**−0.43 (−0.53, −0.32)**	**<0.001**
$100,000 to $199,999	**−0.36 (−0.43, −0.28)**	**<0.001**	**−0.25 (−0.34, −0.16)**	**<0.001**
$200,000 or greater	reference		reference	
Parent’s highest education				
High school education or less	**−0.18 (−0.25, −0.11)**	**<0.001**	**−0.14 (−0.21, −0.07)**	**<0.001**
College education or more	reference		reference	
*R*^2^	0.086	-	0.085	-

**Bold** indicates *p* < 0.05. β=standardized coefficient from linear regression model. *R*^2^ = coefficient of determination from linear regression model. Models represent the abbreviated output from the (1) cross-sectional baseline linear regression models with adjustment for age, sex, sexual orientation, race and ethnicity, household income, parent education, and site, and (2) cross-sectional Year 3 follow-up linear regression models with adjustment for age, sex, sexual orientation, race and ethnicity, household income, parent education, and site. ABCD sampling weights were applied to yield representative estimates based on the American Community Survey from the US Census. MET-h/week scores at baseline and Year 3 follow-up were calculated from lifetime and past year activity involvement, respectively.

## Discussion

### Summary of findings

In this national demographically diverse sample of 9–14-year-old adolescents in the US, our study found that physical activity (MET-h/week) through participation in sports and extracurricular activities and the proportion of early adolescents who met physical activity guidelines decreased over the three years of follow-up. The most common sports and extracurricular activities among early adolescents were soccer, musical instruments, swimming/water polo, baseball/softball, ballet/dance, drawing, and basketball. Notable sex differences included boys being more likely to participate in baseball/softball, basketball, football, martial arts, and soccer, while girls were more likely to engage in ballet/dance, gymnastics, volleyball, drawing/painting, and drama/theater. Additionally, our study shows that household income, parent education, sexual orientation, sex, race and ethnicity, and age were important predictors of sports and extracurricular activity participation levels in early adolescents.

### Time and age effects

Our findings suggest that older age was associated with more physical activity (MET-h/week) through sports and extracurricular activity engagement both at baseline and at Year 3 follow-up. However, the longitudinal analysis showed there was an overall decreasing trend of adolescents who were physically active from Year 1 follow-up to Year 3 follow-up. The reduction in sports and extracurricular activity participation from 2016 to 2021 was potentially due to a pandemic effect, which increased sedentary behaviors due to the closure of school-based physical education facilities, the cancellation of organized sports, and the decrease in unstructured activities.^18^ However, prior literature has also described age-related declines. Several pre-pandemic studies on the trajectories of physical activity have found similar patterns of decreasing levels of physical activity across adolescence,^36,37^ with one study observing a notable decline in sports participation among both males and females during adolescence, especially between the ages of 14 and 18.^38^ This age-related decline may be a result of fewer opportunities for physical activity through sports participation because of school work, employment, and increased social commitments.^23^

### Income and parent education

We found that early adolescents in families with an annual household income of less than $200,000 were more likely to have lower physical activity (MET-h/week) through sports and extracurricular activities compared to those with an annual household income of $200,000 or greater. This finding is consistent with prior studies on physical activity in adolescents and young adults.^23,28^ One underlying factor for the lower physical activity engagement in early adolescents from low-income households may be due to attending underfunded schools that offer fewer intramural activities, after-school sports, and athletic facilities and may have limited access to certified physical education teachers and trainers.^39–44^ One study found that approximately one-third of youth from low-affluence families expressed concerns about getting hurt, feeling unwelcome on teams, and the high expense of sports as key obstacles of sports participation.^28^ Health insurance coverage may also contribute to socioeconomic disparities in physical activity with one study showing that youth with government or no insurance engage in significantly less physical activity compared to those with private insurance.^45^ With public or no health insurance, youth may face difficulty in accessing medical care for pre-participation physical examinations required in many states or rehabilitation for sports injuries,^46^ potentially deterring those from low-income families from returning to physical activities and sports engagement. Adolescents from low-income families are also less likely to receive education about and seek care for sports injuries like concussions,^47,48^ which may inadequately prepare them to manage these injuries. Moreover, adolescents from low-income neighborhoods are less likely to have access to built environment attributes that facilitate higher levels of participation in organized sports, such as recreational facilities, parks, and safety from traffic and crime.^48,49^

Our study also shows that early adolescents with parents who received a high school education or less were also less likely to engage in physical activity through sports and extracurricular activities compared to those with parents who received a college education or more. Using parent education as an indicator of socioeconomic status, one study reveals that adolescents with parents who received a college degree or higher typically reside in neighborhoods with more sports infrastructures, recreational facilities, sidewalks, connected streets, bike trails, and perceived safer outdoor spaces that encourage engagement in physical and sports activity.^50^ Additionally, parents may foster physical activity participation in their children by acting as agents of socialization.^51^ For instance, parents with higher educational attainment may be more aware of the health benefits of sports participation and physical activity^13,43,52,53^ and, thus, are more likely to encourage their children to develop and maintain a more active lifestyle. Prior research highlights that an important predictor of physical and sports activity in adolescents is strong parental support,^31,54^ which encompasses offering encouragement, providing transportation to physical activity and sports opportunities, and participating in sports events with their children.^13,53,55–57^

### Sex and sexual orientation

Similar to previous studies,^23,58^ our study highlights that male early adolescents aged 9–10 years old (baseline) had greater physical activity levels (MET-h/week) and sports and extracurricular activity participation compared to their female counterparts. The lower level of physical activity and sports engagement in female adolescents relative to male adolescents in our study supports the physical activity sex gap.^58^ While male adolescents receive more support for physical activity and physical activity modeling from their peers, female adolescents experience significantly higher levels of perceived barriers to physical and sports activity.^59^ In our study, some activities showed similar participation levels at baseline for both sexes, including lacrosse, skateboarding, and track/running/cross country. However, notable sex differences were observed in activities such as ballet/dance, baseball/softball, football, gymnastics, and volleyball. Exposure to gender norms and existing stereotypes enforce barriers for girls to engage in sports. Some studies report that girls grew concerned that it was ‘not cool’ to play a sport and that sports participation would diminish their femininity.^60^ Similarly, a systematic review of adolescents aged 10–18 years suggests the notion that femininity may decrease sports participation in girls due to concerns about body image, self-esteem, and appearance, which may explain the sex differences in sports and physical activity in our study.^61^ There are also frequent issues with access to sporting opportunities in female sports, which may reduce female sports participation.^60^ Notably, in Year 3 follow-up, sex differences in physical activity (MET-h/week) were not statistically significant, which may be attributed to the overall decreasing trend of physical activity among adolescent participants.

In our study, sexual minority adolescents had lower sports and extracurricular activity in comparison to their heterosexual peers. Prior research similarly found that, in a sample of sexual minority adolescents, more than half of the sample chose not to engage in organized physical activity (i.e., organized sports and group exercises)^62^ since sexual minority adolescents often face several barriers to sports participation. For instance, sexual minority adolescents are more likely to avoid athletic spaces, such as locker rooms, fitness facilities, physical education classes, and sports fields, to prevent feeling unsafe, looking out of place, and bullying and victimization by their peers.^62,63^ In fact, emerging research reveals the high prevalence of homophobia and bullying victimization in team sports and sports clubs drives sexual minority youth to dislike, leave, and choose not to participate in sports and turn to activities that reduce the chances of victimization, such as screen-based activities.^64^ One cross-sectional study found that sexual minority identity was associated with more than three times more hours of recreational screen time than their heterosexual counterparts.^65^ According to the minority stress theory, there are certain social contexts that provide a “safer” environment for sexual minorities than less-safe social spaces.^25^ Screen-based activities can create communities and virtual support for sexual minority youth to connect with others with similar identities and interests. It has been suggested that sexual minority adolescents are more likely to participate in screen-based activities to experience higher levels of social interconnectedness, social support, and feelings of safety, which may contribute to the lower sports participation in sexual minority adolescents.^25^

### Race and ethnicity

Compared to White race at baseline, we found that Asian race was associated with lower physical activity (MET-h/week) through sports and extracurriculars. This was true for soccer, baseball/softball, and ballet/dance, but Asian adolescents had higher odds of participation in activities, including playing musical instruments and swimming/water polo. At Year 3 follow-up, Latino/Hispanic and Native American race and ethnicity were associated with lower sports and extracurricular activity (MET-h/week) compared to White race. Similar to previous studies, our results suggest that disparities in sports participation were present among racial and ethnic minority adolescents.^24^ Lower levels of physical activity through sports participation for racial and ethnic minorities are significantly associated with structural and social determinants.^66^ For example, neighborhoods with higher proportions of Black and Latino/Hispanic residents have less access to gyms and recreational facilities.^67^ Additionally, adolescents aged 12–17 years with better access to multiple parks and recreational facilities near their homes were more likely to partake in organized team sports.^68^ Reduced opportunities for physical and sports activity may also occur due to time constraints, which has been investigated as a common barrier voiced by racial and ethnic minority adolescents.^24^ In one study of low-income racial and ethnic minorities, having other priorities (e.g., work, school, and family) was recognized as a perceived barrier in half of the adolescent focus groups aged 11–15 years.^69^ Priorities that contribute to having less time for physical and sports activity include studying, working a part-time job, or familial obligations like taking care of siblings.^70^ Specifically, Asian adolescents have reported that heavy academic requirements and household chore responsibilities prevent them from participating in physical activity after school,^71^ which may include participation in organized sports. Racial and ethnic differences in physical activity may also be explained by time constraints and scheduling conflicts with parents or caregivers that make it challenging for adolescents to participate in sports.^72^ For example, structured activities often take place after school, when parents may have limited availability to facilitate transportation because of their work schedules. In addition, limited parent support and parental attitudes towards physical activity and sports engagement,^73,74^ lack of social support from peers,^73,75^ negative experiences and lack of motivation, and decreased funding and fewer program opportunities^76^ have also been identified as factors that contribute to the overall decreased physical and sports activity in racial and ethnic minority adolescents.

### Implications

The present study carries significant implications for the education sector, public policy, and clinical practices that can provide insights for targeted interventions. Given the high cost associated with organized sports^43,44^ and that early adolescents already spend the majority of their time at school, school-based physical activity and sports programs may be affordable and accessible venues to promote physical activity, especially for youth from lower socioeconomic status.^77–79^ The education sector should have a vested interest in school-based opportunities such as intramural sports that may not only reduce disparities in sports and physical activity^77–79^ but also help close the achievement gaps through fostering a healthy, active lifestyle that can improve academic performance.^4,80^ Additionally, physical education teachers, trainers, and sports coaches can endorse positive peer relationships and create opportunities for self-exploration.^81^ Fostering a supportive environment may be critical in engaging female and sexual minority adolescents in sports due to the stereotypes and bullying they may face in athletic spaces,^59,60,62,63^ especially those that have been traditionally male-dominated. As for clinical practices, clinicians can address disparities in sports and physical activity by providing education to youth and families about the benefits of an active lifestyle and the preventative measures, care, and return-to-play protocols for sports injuries. Given that sports and physical activity participation in childhood and adolescence is an important predictor of physical activity in adulthood, these types of targeted strategies in early adolescence may aid in the development and maintenance of a healthy and active lifestyle.^82–85^

### Strengths and limitations

Limitations of this study should be noted. Since this is an observational study, causality cannot be determined for our reported associations. This study used parent-reported data to estimate adolescent participation in physical activity. Parent-reported data has proven beneficial in capturing lower physical activity levels, but it has been observed that there are frequent discrepancies between children’s self-reports and their parents’ reports on physical activity.^86^ A study conducted in Norway found that only 5% of parents had accurately estimated their children’s physical activity level, and parents who did not estimate their children’s physical activity level correctly had generally overestimated by three times the amount.^87^ Lastly, the baseline questionnaire assesses lifetime involvement, whereas the follow-up years assess past-year involvement, limiting our ability to directly compare sports and extracurricular activity participation and physical activity level between baseline and follow-up years. As a result, longitudinal analyses were conducted between Year 1 follow-up and Year 3 follow-up only, while cross-sectional analyses comparing baseline and Year 3 follow-up were performed separately.

The strengths of this study include the large, sociodemographically diverse sample of 11,270 early adolescents. In addition, this study uses the SAI-Q, which considers activities that require physical activity beyond sports, such as music and drama, which are typically neglected in other studies. Furthermore, our study uses MET-h/week, which provides a more nuanced understanding of physical activity levels by capturing the intensity and duration of physical activity. Previous studies have generally used physical activity duration or step count, without considering the type and intensity of sports and extracurricular activities.^23,24,26–28,30,88^

## Conclusion

Our findings explore sociodemographic differences in sports and extracurricular activity participation among a diverse population of early adolescents. This analysis revealed activity-specific sociodemographic differences, which can help inform physical activity guidelines. These findings can work to identify effective public health interventions that reduce disparities in physical activity through sports and extracurricular activities and target key populations to implement strategies that acknowledge socioeconomic, sex, sexual orientation, and race and ethnicity differences. Future studies should aim to investigate the mechanisms underlying disparities in physical activity and sports participation and better understand motivations for physical activity among early adolescents.

## Supplementary information

Appendix A

Appendix B

Appendix C

Appendix D

## Data Availability

Data used in the preparation of this article were obtained from the ABCD Study (https://abcdstudy.org), held in the NIMH Data Archive (NDA).

## References

[1] National Research Council (US) and Institute of Medicine (US) Committee on Adolescent Health Care Services and Models of Care for Treatment P, Lawrence, R. S., Gootman, J. A., Sim, L. J. Setting the Stage. In: *Adolescent Health Services: Missing Opportunities*. National Academies Press (US); 2009. Accessed July 19, https://www.ncbi.nlm.nih.gov/books/NBK215426/ (2024). 25009892

[2] Bize, R., Johnson, J. A. & Plotnikoff, R. C. Physical activity level and health-related quality of life in the general adult population: a systematic review. *Prev. Med.* **45**, 401–415 (2007). 17707498 10.1016/j.ypmed.2007.07.017

[3] Dimitri, P., Joshi, K. & Jones, N. Moving Medicine for Children Working Group. Moving more: physical activity and its positive effects on long term conditions in children and young people. *Arch. Dis. Child.* **105**, 1035–1040 (2020). 32198161 10.1136/archdischild-2019-318017

[4] Fox, C. K., Barr-Anderson, D., Neumark-Sztainer, D. & Wall, M. Physical activity and sports team participation: associations with academic outcomes in middle school and high school students. *J. Sch. Health* **80**, 31–37 (2010). 20051088 10.1111/j.1746-1561.2009.00454.x

[5] Nelson, M. C. & Gordon-Larsen, P. Physical activity and sedentary behavior patterns are associated with selected adolescent health risk behaviors. *Pediatrics* **117**, 1281–1290 (2006). 16585325 10.1542/peds.2005-1692

[6] Reiner, M., Niermann, C., Jekauc, D. & Woll, A. Long-term health benefits of physical activity – a systematic review of longitudinal studies. *BMC Public Health* **13**, 813 (2013). 24010994 10.1186/1471-2458-13-813 PMC3847225

[7] Biddle, S. J. H. & Asare, M. Physical activity and mental health in children and adolescents: a review of reviews. *Br. J. Sports Med.* **45**, 886–895 (2011). 21807669 10.1136/bjsports-2011-090185

[8] Sibold, J., Edwards, E., Murray-Close, D. & Hudziak, J. J. Physical activity, sadness, and suicidality in bullied US adolescents. *J. Am. Acad. Child Adolesc. Psychiatry* **54**, 808–815 (2015). 26407490 10.1016/j.jaac.2015.06.019

[9] Fernandes, R. A. & Zanesco, A. Early physical activity promotes lower prevalence of chronic diseases in adulthood. *Hypertens. Res.* **33**, 926–931 (2010). 20574424 10.1038/hr.2010.106

[10] Christofaro, D. G. D. et al. Association of sports practice in childhood and adolescence with cardiac autonomic modulation in adulthood: a retrospective epidemiological study. *Sports Med. Open* **10**, 41 (2024). 38625654 10.1186/s40798-024-00707-7 PMC11021389

[11] Werneck, A. O. et al. Association between sports participation in early life and arterial intima-media thickness among adults. *Medecine* **54**, 85 (2018). 10.3390/medicina54050085 PMC6262615 30428573

[12] Avelar, A. et al. The impact of early sports participation on body fatness in adulthood is not mediated by current physical activity. *Am. J. Hum. Biol.* **36**, e23981 (2024). 37610138 10.1002/ajhb.23981

[13] Logan, K. & Cuff, S. Council on Sports Medicine and Fitness. Organized sports for children, preadolescents, and adolescents. *Pediatrics*. e20190997 10.1542/peds.2019-0997 (2019). 10.1542/peds.2019-0997 31110166

[14] U.S. Department of Health and Human Services. 2018 Physical Activity Guidelines Advisory Committee Scientific Report. 2018. Accessed March 18, 2024. https://health.gov/sites/default/files/2019-09/PAG_Advisory_Committee_Report

[15] World Health Organization. *WHO Guidelines on Physical Activity and Sedentary Behaviour*. World Health Organization; 2020. Accessed July 19, 2024. http://www.ncbi.nlm.nih.gov/books/NBK566045/

[16] Kann, L. et al. Youth Risk Behavior Surveillance—United States, 2017. *MMWR Surveill. Summ.* **67**, 1–114 (2018). 29902162 10.15585/mmwr.ss6708a1 PMC6002027

[17] Guthold, R., Stevens, G. A., Riley, L. M. & Bull, F. C. Global trends in insufficient physical activity among adolescents: a pooled analysis of 298 population-based surveys with 1·6 million participants. *Lancet Child Adolesc. Health* **4**, 23–35 (2020). 31761562 10.1016/S2352-4642(19)30323-2 PMC6919336

[18] Rossi, L., Behme, N. & Breuer, C. Physical activity of children and adolescents during the COVID-19 pandemic-a scoping review. *Int J. Environ. Res. Public Health* **18**, 11440 (2021). 34769956 10.3390/ijerph182111440 PMC8583307

[19] Cortez, C. A. et al. Moderate-to-vigorous intensity physical activity among U.S. adolescents before and during the COVID-19 pandemic: findings from the adolescent brain cognitive development study. *Prev. Med. Rep.* **35**, 102344 (2023). 37564120 10.1016/j.pmedr.2023.102344 PMC10410239

[20] Kawachi, I. Social epidemiology. *Soc. Sci. Med.* **54**, 1739–1741 (2002). 12113431 10.1016/s0277-9536(01)00144-7

[21] Honjo, K. Social epidemiology: definition, history, and research examples. *Environ. Health Prev. Med.* **9**, 193–199 (2004). 21432303 10.1007/BF02898100 PMC2723602

[22] Krieger, N. Theories for social epidemiology in the 21st century: an ecosocial perspective. *Int J. Epidemiol.* **30**, 668–677 (2001). 11511581 10.1093/ije/30.4.668

[23] Armstrong, S. et al. Association of physical activity with income, race/ethnicity, and sex among adolescents and young adults in the United States findings from the National Health and Nutrition Examination Survey, 2007-2016. *JAMA Pediatr.* **172**, 732–740 (2018). 29889945 10.1001/jamapediatrics.2018.1273 PMC6142913

[24] Pontes, N. M. H., Williams, W. M. & Pontes, M. C. F. Interactions between race/ethnicity and gender on physical activity among US high school students: youth risk behavior survey. *J. Pediatr. Nurs.* **60**, 100–108 (2021). 33677258 10.1016/j.pedn.2021.02.013

[25] Perales, F., Campbell, A. & O’Flaherty, M. Sexual orientation and adolescent time use: how sexual minority youth spend their time. *Child Dev.* **91**, 983–1000 (2020). 31062354 10.1111/cdev.13245

[26] Luk, J. W. et al. Sexual minority status and adolescent eating behaviors, physical activity, and weight status. *Am. J. Prev. Med.* **55**, 839–847 (2018). 30344031 10.1016/j.amepre.2018.07.020 PMC6296226

[27] Mereish, E. H. & Poteat, V. P. Let’s get physical: sexual orientation disparities in physical activity, sports involvement, and obesity among a population-based sample of adolescents. *Am. J. Public Health* **105**, 1842–1848 (2015). 26180946 10.2105/AJPH.2015.302682 PMC4539805

[28] Tandon, P. S. et al. Socioeconomic inequities in youth participation in physical activity and sports. *Int. J. Environ. Res. Public Health* **18**, 6946 (2021). 34209544 10.3390/ijerph18136946 PMC8297079

[29] Scholes, S. & Mindell, J. S. Income-based inequalities in self-reported moderate-to-vigorous physical activity among adolescents in England and the USA: a cross-sectional study. *BMJ Open* **11**, e040540 (2021). 33589448 10.1136/bmjopen-2020-040540 PMC7887356

[30] Ke, Y. et al. Associations between socioeconomic status and physical activity: a cross-sectional analysis of Chinese children and adolescents. *Front. Psychol.* **13**, 904506 (2022). 36118481 10.3389/fpsyg.2022.904506 PMC9477139

[31] Sterdt, E., Liersch, S. & Walter, U. Correlates of physical activity of children and adolescents: a systematic review of reviews. *Health Educ. J.* **73**, 72–89 (2014).

[32] Ruedl, G. et al. Impact of parental education and physical activity on the long-term development of the physical fitness of primary school children: an observational study. *Int. J. Environ. Res. Public Health* **18**, 8736 (2021). 34444484 10.3390/ijerph18168736 PMC8391261

[33] Huppertz, C. et al. Individual differences in exercise behavior: stability and change in genetic and environmental determinants from age 7 to 18. *Behav. Genet* **46**, 665–679 (2016). 27406597 10.1007/s10519-016-9799-x

[34] Butte, N. F. et al. A youth compendium of physical activities: activity codes and metabolic intensities. *Med. Sci. Sports Exerc.* **50**, 246–256 (2018). 28938248 10.1249/MSS.0000000000001430 PMC5768467

[35] Ainsworth, B. E. et al. Compendium of physical activities: an update of activity codes and MET intensities. *Med. Sci. Sports Exerc.* **32**, 498 (2000). 10.1097/00005768-200009001-00009 10993420

[36] Janz, K. F. et al. Objectively measured physical activity trajectories predict adolescent bone strength: Iowa Bone Development Study. *Br. J. Sports Med.* **48**, 1032–1036 (2014). 24837241 10.1136/bjsports-2014-093574 PMC4550443

[37] Lounassalo, I. et al. Distinct trajectories of physical activity and related factors during the life course in the general population: a systematic review. *BMC Public Health* **19**, 271 (2019). 30841921 10.1186/s12889-019-6513-y PMC6404287

[38] Emmonds, S., Till, K., Weaving, D., Burton, A. & Lara-Bercial, S. Youth sport participation trends across Europe: implications for policy and practice. *Res. Q Exerc. Sport* **95**, 69–80 (2024). 36697376 10.1080/02701367.2022.2148623

[39] Morin, P., Lebel, A., Robitaille, É & Bisset, S. Socioeconomic factors influence physical activity and sport in Quebec schools. *J. Sch. Health* **86**, 841–851 (2016). 27714872 10.1111/josh.12438

[40] Carlson, J. A. et al. Socioeconomic disparities in elementary school practices and children’s physical activity during school. *Am. J. Health Promot.* **28**, S47–S53 (2014). 24380465 10.4278/ajhp.130430-QUAN-206 PMC4082956

[41] Young, D. R. et al. Policies and opportunities for physical activity in middle school environments. *J. Sch. Health* **77**, 41–47 (2007). 17212759 10.1111/j.1746-1561.2007.00161.x PMC2475674

[42] Post, E. et al. School and community socioeconomic status and access to athletic trainer services in Wisconsin secondary schools. *J. Athl. Train.* **54**, 177–181 (2019). 30398929 10.4085/1062-6050-440-17 PMC6464297

[43] Stalsberg, R. & Pedersen, A. V. Effects of socioeconomic status on the physical activity in adolescents: a systematic review of the evidence. *Scand. J. Med. Sci. Sports* **20**, 368–383 (2010). 20136763 10.1111/j.1600-0838.2009.01047.x

[44] Romero, A. J. Low-income neighborhood barriers and resources for adolescents’ physical activity. *J. Adolesc. Health* **36**, 253–259 (2005). 15737782 10.1016/j.jadohealth.2004.02.027

[45] Fabricant, P. D. et al. Association between government health insurance status and physical activity in American youth. *J. Pediatr. Orthop.* **39**, e552–e557 (2019). 30624341 10.1097/BPO.0000000000001329

[46] Wiznia, D. H. et al. The influence of medical insurance on patient access to orthopaedic surgery sports medicine appointments under the Affordable Care Act. *Orthop. J. Sports Med.* **5**, 2325967117714140 (2017). 28812034 10.1177/2325967117714140 PMC5528187

[47] Donnell, Z., Hoffman, R., Sarmiento, K. & Hays, C. Concussion attitudes, behaviors, and education among youth ages 12-17: results from the 2014 YouthStyles survey. *J. Saf. Res.* **64**, 163–169 (2018). 10.1016/j.jsr.2017.12.001 PMC6207930 29636165

[48] Turner, R. W., Lucas, J. W., Margolis, L. H. & Corwell, B. N. A preliminary study of youth sport concussions: parents’ health literacy and knowledge of return-to-play protocol criteria. *Brain Inj.* **31**, 1124–1130 (2017). 28506094 10.1080/02699052.2017.1298003 PMC6193450

[49] Heradstveit, O. et al. Physical inactivity, non-participation in sports and socioeconomic status: a large population-based study among Norwegian adolescents. *BMC Public Health* **20**, 1010 (2020). 32590961 10.1186/s12889-020-09141-2 PMC7318733

[50] Gordon-Larsen, P., Nelson, M. C., Page, P. & Popkin, B. M. Inequality in the built environment underlies key health disparities in physical activity and obesity. *Pediatrics* **117**, 417–424 (2006). 16452361 10.1542/peds.2005-0058

[51] Pugliese, J. & Tinsley, B. Parental socialization of child and adolescent physical activity: a meta-analysis. *J. Fam. Psychol.* **21**, 331–343 (2007). 17874918 10.1037/0893-3200.21.3.331

[52] Sekulic, D., Rodek, J. & Sattler, T. Factors associated with physical activity levels in late adolescence: a prospective study. *Med. Pr.* **71**, 637–647 (2020). 32870182 10.13075/mp.5893.01012

[53] Eime, R. M. Family support and ease of access link socio-economic status and sports club membership in adolescent girls: a mediation study. *Int J. Behav. Nutr. Phys. Act.* **10**, 50 (2013). 23618407 10.1186/1479-5868-10-50 PMC3639833

[54] Hu, D., Zhou, S., Crowley-McHattan, Z. J. & Liu, Z. Factors that influence participation in physical activity in school-aged children and adolescents: a systematic review from the social ecological model perspective. *Int J. Environ. Res. Public Health* **18**, 3147 (2021). 33803733 10.3390/ijerph18063147 PMC8003258

[55] Beets, M. W. et al. Social support and youth physical activity: the role of provider and type. *Am. J. Health Behav.* **30**, 278–289 (2006). 16712442 10.5555/ajhb.2006.30.3.278

[56] Duncan, S. C., Duncan, T. E. & Strycker, L. A. Sources and types of social support in youth physical activity. *Health Psychol.* **24**, 3–10 (2005). 15631557 10.1037/0278-6133.24.1.3

[57] Trost, S. G. & Loprinzi, P. D. Parental influences on physical activity behavior in children and adolescents: a brief review. *Am. J. Lifestyle Med.* **5**, 171–181 (2011).

[58] Luque-Casado, A., Mayo, X., Lavín-Pérez, A. M., Jiménez, A. & Del Villar, F. Understanding behavioral regulation towards physical activity participation: do we need a paradigm shift to close the gender gap?. *Sustainability* **13**, 1683 (2021).

[59] Sallis, J. F., Zakarian, J. M., Hovell, M. F. & Hofstetter, C. R. Ethnic, socioeconomic, and sex differences in physical activity among adolescents. *J. Clin. Epidemiol.* **49**, 125–134 (1996). 8606313 10.1016/0895-4356(95)00514-5

[60] Slater, A. & Tiggemann, M. Uncool to do sport”: a focus group study of adolescent girls’ reasons for withdrawing from physical activity. *Psychol. Sport Exerc.* **11**, 619–626 (2010).

[61] Gualdi-Russo, E., Rinaldo, N. & Zaccagni, L. Physical activity and body image perception in adolescents: a systematic review. *Int J. Environ. Res. Public Health* **19**, 13190 (2022). 36293770 10.3390/ijerph192013190 PMC9603811

[62] Parchem, B. et al. Barriers to participation in organized physical activity among LGBTQ+ youth: differences by sexual, gender, and racial identities. *J. Phys. Act. Health* **21**, 698–706 (2024). 38626889 10.1123/jpah.2023-0652

[63] Greenspan, S. B., Griffith, C. & Watson, R. J. LGBTQ+ youth’s experiences and engagement in physical activity: a comprehensive content analysis. *Adolesc. Res. Rev.* **4**, 169–185 (2019).

[64] Osborne, D. & Wagner, W. E. Exploring the relationship between homophobia and participation in core sports among high school students. *Sociol. Perspect.* **50**, 597–613 (2007).

[65] Nagata, J. M. et al. Associations between sexual orientation and early adolescent screen use: findings from the Adolescent Brain Cognitive Development (ABCD) Study. *Ann. Epidemiol.* **82**, 54–58.e1 (2023). 36965838 10.1016/j.annepidem.2023.03.004 PMC10793659

[66] Cheng, G. J. & Nicklett, E. J. Racial and ethnic differences in the relationship between neighborhood environment and physical activity among middle-aged and older adults. *J. Aging Health* **34**, 1163–1177 (2022). 35603774 10.1177/08982643221103359

[67] Watson K. B. Disparities in adolescents’ residence in neighborhoods supportive of physical activity — United States, 2011–2012. *MMWR Morb Mortal Wkly Rep*. **65**, 10.15585/mmwr.mm6523a2 (2016). 10.15585/mmwr.mm6523a2 27309671

[68] McCormack M. et al. Availability of recreation facilities and parks in relation to adolescent participation in organized sports and activity programs. *J. Healthy Eat Act. Living*. **3**, 19–35 (2023) PMC10546936 37794920

[69] Bragg, M. A., Tucker, C. M., Kaye, L. B. & Desmond, F. Motivators of and barriers to engaging in physical activity: perspectives of low-income culturally diverse adolescents and adults. *Am. J. Health Educ.* **40**, 146–154 (2009). 29527247 10.1080/19325037.2009.10599089 PMC5844488

[70] Martins, J., Marques, A., Sarmento, H. & Carreiro da Costa, F. Adolescents’ perspectives on the barriers and facilitators of physical activity: a systematic review of qualitative studies. *Health Educ. Res.* **30**, 742–755 (2015). 26324394 10.1093/her/cyv042

[71] Dave, S. S. et al. Life stage influences on U.S. South Asian women’s physical activity. *Am. J. Health Promot* **29**, e100–e108 (2015). 24717067 10.4278/ajhp.130415-QUAL-175

[72] Finkelstein, D. M., Petersen, D. M. & Schottenfeld, L. S. Promoting children’s physical activity in low-income communities in Colorado: what are the barriers and opportunities?. *Prevent. Chronic Dis.* **14**, 170111 (2017). 10.5888/pcd14.170111 PMC5737980 29240553

[73] Prochaska, J. J., Rodgers, M. W. & Sallis, J. F. Association of parent and peer support with adolescent physical activity. *Res. Q Exerc. Sport* **73**, 206–210 (2002). 12092896 10.1080/02701367.2002.10609010

[74] Bauer, K. W., Nelson, M. C., Boutelle, K. N. & Neumark-Sztainer, D. Parental influences on adolescents’ physical activity and sedentary behavior: longitudinal findings from Project EAT-II. *Int. J. Behav. Nutr. Phys. Act.* **5**, 12 (2008). 18302765 10.1186/1479-5868-5-12 PMC2265744

[75] Eyler, A. A. et al. Physical activity social support and middle- and older-aged minority women: results from a US survey. *Soc. Sci. Med.* **49**, 781–789 (1999). 10459889 10.1016/s0277-9536(99)00137-9

[76] National Women’s Law Center [NWLC] and the Poverty & Race Research Action Council [PRRAC]. *Finishing Last: Girls of Color and School Sports Opportunities*. (National Women’s Law Center [NWLC] and the Poverty & Race Research Action Council [PRRAC], 2015). https://nwlc.org/wp-content/uploads/2022/03/final_nwlc_girlsfinishinglast_report.pdf

[77] Kanters, M. A. et al. School sport participation under two school sport policies: comparisons by race/ethnicity, gender, and socioeconomic status. *Ann. Behav. Med.* **45**, S113–S121 (2013). 22993023 10.1007/s12160-012-9413-2

[78] De Meester, A. et al. Extracurricular school-based sports as a motivating vehicle for sports participation in youth: a cross-sectional study. *Int. J. Behav. Nutr. Phys. Act.* **11**, 48 (2014). 24708585 10.1186/1479-5868-11-48 PMC4233643

[79] Sallis, J. F. et al. Environmental interventions for eating and physical activity: a randomized controlled trial in middle schools. *Am. J. Prev. Med.* **24**, 209–217 (2003). 12657338 10.1016/s0749-3797(02)00646-3

[80] Basch, C. E. Healthier students are better learners: high-quality, strategically planned, and effectively coordinated school health programs must be a fundamental mission of schools to help close the achievement gap. *J. Sch. Health* **81**, 650–662 (2011). 21923878 10.1111/j.1746-1561.2011.00640.x

[81] Fuller, R. D., Percy, V. E., Bruening, J. E. & Cotrufo, R. J. Positive youth development: minority male participation in a sport-based afterschool program in an urban environment. *Res. Q Exerc. Sport* **84**, 469–482 (2013). 24592777 10.1080/02701367.2013.839025

[82] Miller, K., Morley, C., Fraser, B. J., Gall, S. L. & Cleland, V. Types of leisure-time physical activity participation in childhood and adolescence, and physical activity behaviours and health outcomes in adulthood: a systematic review. *BMC Public Health* **24**, 1789 (2024). 38965532 10.1186/s12889-024-19050-3 PMC11225122

[83] Telama, R., Yang, X., Laakso, L. & Viikari, J. Physical activity in childhood and adolescence as predictor of physical activity in young adulthood. *Am. J. Prev. Med.* **13**, 317–323 (1997). 9236971

[84] Batista, M. B. et al. Sports participation in childhood and adolescence and physical activity intensity in adulthood. *PLoS ONE* **19**, e0299604 (2024). 38696508 10.1371/journal.pone.0299604 PMC11065273

[85] Kjønniksen, L., Anderssen, N. & Wold, B. Organized youth sport as a predictor of physical activity in adulthood. *Scand. J. Med. Sci. Sports* **19**, 646–654 (2009). 18694430 10.1111/j.1600-0838.2008.00850.x

[86] Nagata, J. M. et al. Parent-adolescent agreement in reported moderate-to-vigorous intensity physical activity during the COVID-19 pandemic. *BMC Public Health* **22**, 332 (2022). 35172771 10.1186/s12889-022-12530-4 PMC8851835

[87] Kippe, K., Marques, A., Martins, J. & Lagestad, P. A. Parents’ inadequate estimate of their children’s objectively physical activity level. *Childhood* **9**, 392 (2022). 10.3390/children9030392 PMC8947066 35327764

[88] Nagata, J. M. et al. Social epidemiology of Fitbit daily steps in early adolescence. *Pediatr. Res.* **94**, 1838–1844 (2023). 37353663 10.1038/s41390-023-02700-4 PMC10624619

