# Supplementary material for: Social epidemiology of sports and extracurricular activities in early adolescents
Source: Pediatr Res. 2025 May 4;98(4):1313–22. doi: 10.1038/s41390-025-04099-6 (PMC12326775; doi:10.1038/s41390-025-04099-6)
Supplement: Supplementary file 1 — Appendix A [file 41390_2025_4099_MOESM1_ESM.pdf]

Appendix A. Comparison of the sociodemographic characteristics of the Adolescent Brain Cognitive Development (ABCD) study participants included vs. excluded in the analysis

Sociodemographic characteristics	Included (n=11,270)	Excluded (n=692)	p
Age (years)	9.9 (0.6)	10.0 (0.6)	0.096
Biological sex at birth			0.576
Female	48.8%	50.1%	
Male	51.2%	49.9%	
Sexual orientation (Year 3)			0.009
Heterosexual	82.5%	84.1%	
Maybe gay/bisexual	5.2%	2.7%	
Gay/bisexual	8.3%	6.9%	
Don't understand the question	2.5%	4.5%	
Refuse to answer	1.6%	1.8%	
Race and ethnicity			<0.001
Asian	5.5%	5.7%	
Black	16.6%	30.8%	
Latino/Hispanic	19.7%	28.8%	
Native American	3.2%	3.6%	
Other	1.4%	1.7%	
White	53.6%	29.4%	
Household income			<0.001
$24,999 or less	18.3%	47.1%	
$25,000 to $49,999	20.3%	28.3%	
$50,000 to $74,999	17.6%	9.5%	
$75,000 to $99,999	13.5%	3.8%	
$100,000 to $199,999	22.8%	10.7%	
$200,000 or greater	7.5%	0.6%	
Parent's highest education			<0.001
High school education or less	18.9%	45.4%	
College education or more	81.1%	54.6%	
