# Supplementary material for: Social epidemiology of sports and extracurricular activities in early adolescents
Source: Pediatr Res. 2025 May 4;98(4):1313–22. doi: 10.1038/s41390-025-04099-6 (PMC12326775; doi:10.1038/s41390-025-04099-6)
Supplement: Supplementary file 2 — Appendix B [file 41390_2025_4099_MOESM2_ESM.pdf]

Appendix B. Prevalence of participation in each sport and extracurricular activity by sex from baseline to year 3

Activity	Baseline (lifetime prevalence)		Year 1 (since last visit)		Year 2 (since last visit)		Year 3 (since last visit)	
	Male	Female	Male	Female	Male	Female	Male	Female
Ballet, dance	4.1%	45.6%	2.6%	19.2%	1.9%	15.2%	1.2%	12.0%
Baseball, softball	35.6%	15.7%	18.6%	8.2%	14.9%	6.6%	11.5%	5.1%
Basketball	33.1%	14.8%	25.0%	12.6%	21.7%	11.7%	18.0%	8.7%
Climbing	3.2%	3.2%	2.8%	2.3%	2.0%	1.4%	1.4%	0.9%
Field hockey	0.4%	0.7%	0.4%	0.9%	0.3%	1.1%	0.2%	1.1%
Football	19.7%	1.5%	15.1%	0.9%	13.2%	0.7%	10.6%	0.6%
Gymnastics	7.5%	35.5%	1.9%	14.0%	1.1%	8.8%	0.9%	5.3%
Ice hockey	3.3%	1.2%	2.2%	0.7%	2.0%	0.8%	2.1%	0.7%
Horseback riding, polo	1.9%	5.8%	0.9%	3.1%	0.7%	2.4%	0.6%	2.3%
Ice or inline skating	3.9%	7.8%	1.8%	4.1%	1.0%	2.1%	1.0%	1.4%
Martial arts	22.1%	11.9%	8.5%	4.8%	5.9%	3.7%	4.7%	2.8%
Lacrosse	2.7%	2.0%	2.4%	1.9%	2.2%	1.8%	1.6%	1.5%
Rugby	0.4%	0.1%	0.4%	0.1%	0.2%	0.0%	0.3%	0.1%
Skateboarding	5.6%	2.4%	4.2%	1.6%	4.4%	2.7%	4.3%	4.1%
Skiing, snowboarding	6.5%	5.9%	5.4%	4.4%	5.6%	3.9%	5.3%	3.7%
Soccer	45.2%	31.0%	26.2%	16.7%	19.8%	13.8%	15.5%	10.4%
Surfing	0.4%	0.5%	0.5%	0.3%	0.3%	0.2%	0.5%	0.4%
Swimming	28.7%	32.3%	18.0%	19.9%	11.4%	13.1%	8.1%	8.8%
Tennis	6.1%	5.8%	3.8%	3.1%	3.7%	2.8%	3.4%	2.6%
Track, running, cross-country	5.3%	7.2%	5.5%	7.7%	6.3%	6.6%	5.9%	6.2%
Wrestling, mixed martial arts	4.7%	1.0%	2.7%	0.5%	2.5%	0.5%	2.5%	0.4%
Volleyball	0.6%	4.6%	0.9%	6.3%	1.0%	8.4%	1.3%	8.8%
Yoga, tai chi	1.2%	3.4%	0.7%	1.7%	0.4%	1.2%	0.3%	1.3%
Musical instrument (singing, choir, guitar, piano, drums, violin, flute, band, rock band, orchestra)	32.7%	41.7%	34.3%	40.6%	33.9%	37.0%	28.4%	30.7%
Drawing, painting, graphic art, photography, pottery, sculpting	14.6%	26.4%	13.2%	25.6%	9.8%	21.5%	8.6%	20.2%
Drama, theater, acting, film	6.7%	14.0%	5.6%	12.9%	5.5%	12.1%	3.8%	9.5%
Crafts like knitting, building model cars of airplanes	6.3%	11.0%	5.4%	9.8%	3.1%	7.3%	2.3%	6.6%
Competitive games like chess, cards, or darts	13.0%	7.4%	10.5%	5.9%	7.1%	3.6%	5.9%	2.8%
Hobbies like collecting stamps or coins	8.3%	5.3%	5.4%	3.7%	3.9%	2.2%	2.8%	1.6%
My child has not participated in any of the above activities	11.9%	12.7%	17.7%	16.3%	22.3%	20.4%	27.7%	27.2%

Propensity weights were applied to yield representative estimates based on the American Community Survey from the US Census. MET-h/week scores at baseline and follow-up years were calculated from lifetime and past year activity involvement, respectively.
