# Supplementary material for: Social epidemiology of sports and extracurricular activities in early adolescents
Source: Pediatr Res. 2025 May 4;98(4):1313–22. doi: 10.1038/s41390-025-04099-6 (PMC12326775; doi:10.1038/s41390-025-04099-6)
Supplement: Supplementary file 3 — Appendix C [file 41390_2025_4099_MOESM3_ESM.pdf]

Appendix C. Prevalence of participation in each sport and extracurricular activity by race and ethnicity from baseline to year 3

	Baseline (lifetime)						Year 1 (since last visit)						Year 2 (since last visit)						Year 3 (since last visit)					
Activity	Asian	Black	Latino/ Hispanic	Native American	Other	White	Asian	Black	Latino/ Hispanic	Native American	Other	White	Asian	Black	Latino/ Hispanic	Native American	Other	White	Asian	Black	Latino/ Hispanic	Native American	Other	White
Ballet, dance	24.9%	22.0%	22.6%	17.0%	31.1%	25.9%	12.4%	13.1%	11.5%	11.0%	11.6%	9.5%	8.7%	10.5%	8.6%	8.0%	8.1%	7.6%	7.8%	7.0%	6.3%	3.2%	7.8%	6.3%
Baseball, softball	16.3%	14.2%	15.9%	26.2%	21.0%	34.2%	8.8%	7.8%	8.1%	6.4%	13.9%	18.0%	5.6%	5.2%	7.0%	6.6%	9.1%	14.8%	5.0%	3.9%	5.9%	5.4%	6.0%	11.0%
Basketball	22.2%	28.1%	16.0%	20.4%	26.9%	26.3%	18.8%	25.1%	13.4%	12.4%	23.5%	19.4%	10.9%	24.2%	10.6%	15.5%	23.4%	17.4%	8.7%	20.1%	9.5%	8.3%	15.3%	13.8%
Climbing	4.0%	3.0%	1.7%	3.0%	2.0%	3.7%	4.0%	2.8%	1.9%	1.8%	3.8%	2.6%	2.3%	1.8%	0.8%	1.8%	2.0%	1.9%	0.7%	1.0%	0.6%	1.0%	1.9%	1.4%
Field hockey	0.4%	0.1%	0.2%	0.0%	0.2%	0.9%	1.0%	0.2%	0.2%	0.0%	0.2%	0.9%	0.2%	0.2%	0.2%	0.1%	1.0%	1.0%	0.2%	0.3%	0.4%	0.0%	0.0%	0.9%
Football	5.7%	19.9%	6.7%	10.2%	14.2%	10.0%	3.3%	15.3%	5.9%	6.4%	13.7%	7.4%	2.0%	13.4%	4.8%	6.2%	11.3%	6.6%	1.6%	10.5%	4.8%	4.2%	8.8%	5.1%
Gymnastics	23.1%	12.0%	13.4%	20.9%	17.6%	26.7%	6.7%	6.1%	6.6%	8.9%	10.6%	8.6%	3.2%	4.1%	4.0%	5.1%	3.5%	5.5%	1.8%	2.5%	2.2%	4.0%	5.4%	3.5%
Ice hockey	1.4%	0.2%	1.1%	0.6%	0.8%	3.6%	0.5%	0.0%	0.6%	0.3%	0.7%	2.4%	0.7%	0.1%	0.8%	1.4%	0.7%	2.1%	0.4%	0.2%	0.7%	0.7%	0.7%	2.1%
Horseback riding, polo	3.5%	1.6%	2.8%	2.9%	4.4%	4.9%	1.7%	0.7%	1.1%	0.7%	3.9%	2.7%	0.6%	0.7%	0.6%	0.3%	2.1%	2.3%	1.2%	0.6%	0.7%	0.7%	2.3%	2.0%
Ice or inline skating	11.5%	2.8%	4.6%	3.4%	2.1%	6.8%	5.8%	2.0%	2.1%	0.8%	4.8%	3.3%	4.1%	0.5%	1.2%	1.3%	3.1%	1.6%	1.0%	0.8%	1.2%	0.7%	2.2%	1.2%
Martial arts	24.4%	10.4%	17.0%	15.1%	16.6%	18.6%	13.3%	4.8%	6.6%	5.9%	3.8%	6.7%	9.2%	3.2%	5.3%	4.9%	2.0%	4.8%	7.5%	2.0%	3.6%	4.6%	0.9%	4.0%
Lacrosse	1.9%	0.9%	0.9%	0.5%	1.6%	3.5%	1.2%	0.9%	0.7%	0.9%	1.9%	3.2%	0.7%	0.7%	0.8%	0.3%	1.9%	3.0%	0.8%	0.4%	0.7%	0.2%	0.0%	2.4%
Rugby	1.1%	0.1%	0.3%	0.1%	0.0%	0.3%	1.2%	0.2%	0.2%	0.1%	0.0%	0.2%	0.5%	0.1%	0.0%	0.0%	0.0%	0.1%	1.2%	0.2%	0.1%	0.0%	0.0%	0.1%
Skateboarding	3.6%	3.1%	5.0%	3.9%	7.3%	3.9%	2.0%	3.4%	3.2%	4.1%	3.7%	2.7%	2.3%	3.0%	3.3%	6.0%	3.2%	3.9%	2.5%	3.9%	4.5%	5.3%	14.8%	4.0%
Skiing, snowboarding	6.0%	0.9%	1.5%	2.0%	2.9%	9.9%	5.0%	0.3%	0.8%	1.9%	0.6%	8.0%	4.1%	0.5%	1.2%	2.0%	1.3%	7.5%	4.0%	0.7%	0.8%	1.6%	0.8%	7.2%
Soccer	34.9%	18.5%	34.0%	29.8%	36.8%	46.9%	23.0%	9.6%	23.6%	16.1%	27.3%	24.4%	21.7%	7.5%	16.7%	15.1%	15.0%	19.3%	14.6%	5.3%	12.7%	10.3%	14.7%	15.2%
Surfing	0.3%	0.1%	0.4%	0.3%	1.0%	0.6%	0.2%	0.2%	0.4%	0.1%	0.0%	0.5%	0.4%	0.2%	0.2%	0.6%	0.0%	0.3%	0.2%	0.2%	0.5%	0.0%	0.0%	0.5%
Swimming	45.0%	23.1%	25.0%	20.9%	33.6%	33.8%	26.0%	19.6%	15.5%	13.3%	30.7%	19.2%	24.4%	10.0%	8.1%	11.6%	10.4%	13.2%	11.4%	7.4%	6.7%	5.7%	18.4%	8.9%
Tennis	11.1%	2.4%	5.0%	1.8%	3.7%	7.2%	7.9%	1.4%	2.5%	1.6%	3.7%	4.0%	9.2%	1.4%	2.2%	0.8%	0.0%	3.8%	8.0%	1.5%	2.3%	0.6%	0.2%	3.4%
Track, running, cross-country	5.1%	6.3%	3.7%	5.5%	3.6%	7.4%	7.6%	6.0%	4.4%	3.8%	6.5%	7.5%	8.2%	4.8%	3.1%	6.0%	6.9%	7.9%	6.0%	4.9%	3.2%	7.2%	3.3%	7.3%
Wrestling, mixed martial arts	1.3%	2.4%	3.0%	2.4%	3.2%	3.1%	1.2%	1.5%	2.1%	2.6%	1.9%	1.5%	2.1%	1.5%	1.2%	0.7%	1.7%	1.6%	1.1%	1.1%	1.5%	1.7%	2.1%	1.6%
Volleyball	2.0%	1.9%	2.1%	2.4%	1.8%	3.0%	3.1%	2.4%	2.8%	3.1%	1.6%	4.2%	5.4%	2.4%	3.3%	6.0%	5.1%	5.6%	5.6%	3.8%	4.1%	5.1%	3.3%	5.4%
Yoga, tai chi	3.6%	1.9%	2.2%	1.5%	5.6%	2.2%	0.2%	1.9%	1.3%	0.8%	6.7%	1.0%	0.7%	1.0%	0.6%	0.5%	0.0%	0.8%	0.6%	0.8%	0.5%	0.8%	1.0%	0.9%
Musical instrument (singing, choir, guitar, piano, drums, violin, flute, band, rock band, orchestra)	56.1%	25.4%	26.6%	25.8%	34.9%	43.4%	48.8%	24.0%	25.7%	28.0%	38.8%	44.7%	48.6%	18.7%	22.8%	24.6%	36.8%	43.9%	39.7%	14.5%	16.8%	24.8%	23.3%	37.5%
Drawing, painting, graphic art, photography, pottery, sculpting	27.8%	16.6%	17.7%	27.7%	25.0%	21.1%	19.1%	16.0%	18.2%	26.6%	28.7%	19.9%	15.4%	12.0%	13.7%	17.2%	22.2%	16.9%	12.6%	10.5%	11.8%	18.2%	18.0%	16.0%
Drama, theater, acting, film	13.0%	6.6%	8.0%	10.7%	14.2%	11.8%	7.4%	4.9%	6.8%	11.2%	8.7%	11.2%	6.0%	4.4%	5.5%	8.9%	6.2%	11.4%	3.9%	4.2%	3.4%	7.8%	9.8%	8.5%
Crafts like knitting, building model cars of airplanes	9.1%	5.4%	5.6%	9.6%	14.5%	10.3%	8.1%	4.3%	6.0%	8.5%	11.5%	8.8%	5.9%	2.8%	3.5%	6.4%	6.2%	6.2%	5.1%	2.1%	3.1%	5.3%	6.1%	5.3%
Competitive games like chess, cards, or darts	14.1%	6.6%	6.8%	9.8%	10.1%	12.4%	9.9%	6.8%	5.8%	6.0%	14.2%	9.4%	6.1%	3.2%	4.4%	6.7%	7.4%	6.2%	4.6%	3.2%	3.4%	4.3%	10.3%	4.9%
Hobbies like collecting stamps or coins	6.6%	3.7%	6.7%	9.9%	8.1%	7.7%	4.8%	2.0%	4.6%	5.1%	5.7%	5.2%	3.1%	1.3%	2.8%	5.0%	1.9%	3.7%	2.4%	0.8%	1.4%	3.4%	3.5%	2.8%
My child has not participated in any of the above activities	8.4%	23.8%	17.4%	16.1%	14.1%	7.0%	11.4%	27.7%	22.9%	26.0%	18.3%	11.8%	14.6%	34.6%	33.3%	25.6%	24.4%	13.7%	21.5%	41.7%	40.4%	37.2%	22.2%	19.1%

Propensity weights were applied to yield representative estimates based on the American Community Survey from the US Census. MET-h/week scores at baseline and follow-up years were calculated from lifetime and past year activity involvement, respectively.
