# Supplementary material for: Social epidemiology of sports and extracurricular activities in early adolescents
Source: Pediatr Res. 2025 May 4;98(4):1313–22. doi: 10.1038/s41390-025-04099-6 (PMC12326775; doi:10.1038/s41390-025-04099-6)
Supplement: Supplementary file 4 — Appendix D [file 41390_2025_4099_MOESM4_ESM.pdf]

Appendix D. Sociodemographic associations with lifetime involvement in the most common sports and extracurricular activities at baseline in the Adolescent Brain Cognitive Development (ABCD) Study

	Soccer		Musical instrument		Swimming, water polo		Baseball, softball		Ballet, dance	
Sociodemographic characteristics	Adjusted OR (95% CI)	p	Adjusted OR (95% CI)	p	Adjusted OR (95% CI)	p	Adjusted OR (95% CI)	p	Adjusted OR (95% CI)	p
Age	1.04 (0.97, 1.12)	0.285	**1.54 (1.42, 1.66)**	**<0.001**	1.04 (0.96, 1.12)	0.353	1.08 (0.99, 1.17)	0.075	1.07 (0.98, 1.18)	0.142
Biological sex at birth										
Female	reference		reference		reference		reference		reference	
Male	**1.84 (1.67, 2.03)**	**<0.001**	**0.63 (0.57, 0.69)**	**<0.001**	**0.81 (0.74, 0.90)**	**<0.001**	**3.21 (2.87, 3.58)**	**<0.001**	**0.04 (0.04, 0.05)**	**<0.001**
Sexual orientation (Year 3)										
Heterosexual	reference		reference		reference		reference		reference	
Maybe gay/bisexual	**0.77 (0.62, 0.96)**	**0.020**	1.17 (0.95, 1.44)	0.144	0.91 (0.74, 1.12)	0.396	**0.67 (0.51, 0.88)**	**0.004**	0.93 (0.74, 1.16)	0.497
Gay/bisexual	**0.74 (0.62, 0.90)**	**0.002**	1.05 (0.88, 1.26)	0.585	0.95 (0.79, 1.14)	0.553	0.87 (0.69, 1.09)	0.222	0.89 (0.74, 1.07)	0.233
Don't understand the question	1.05 (0.79, 1.41)	0.725	1.07 (0.78, 1.45)	0.686	1.14 (0.85, 1.52)	0.385	**0.68 (0.48, 0.97)**	**0.032**	0.85 (0.60, 1.20)	0.360
Refuse to answer	0.75 (0.50, 1.11)	0.144	1.00 (0.68, 1.46)	0.986	1.23 (0.84, 1.80)	0.286	**0.45 (0.27, 0.73)**	**0.001**	0.96 (0.63, 1.47)	0.861
Race and ethnicity										
Asian	**0.54 (0.43, 0.67)**	**<0.001**	**1.57 (1.24, 1.99)**	**<0.001**	**1.37 (1.09, 1.72)**	**0.007**	**0.35 (0.26, 0.46)**	**<0.001**	**0.74 (0.55, 0.98)**	**0.037**
Black	**0.46 (0.39, 0.54)**	**<0.001**	**0.78 (0.67, 0.90)**	**0.001**	0.89 (0.76, 1.03)	0.114	**0.48 (0.40, 0.57)**	**<0.001**	1.11 (0.92, 1.33)	0.260
Latino / Hispanic	0.97 (0.82, 1.13)	0.666	**0.78 (0.66, 0.93)**	**0.004**	0.88 (0.75, 1.04)	0.138	**0.69 (0.57, 0.83)**	**<0.001**	1.04 (0.84, 1.28)	0.734
Native American	0.75 (0.56, 1.02)	0.067	0.83 (0.60, 1.16)	0.285	0.81 (0.58, 1.13)	0.214	1.06 (0.76, 1.47)	0.747	0.71 (0.48, 1.06)	0.091
Other	0.95 (0.57, 1.59)	0.847	0.97 (0.58, 1.63)	0.917	1.35 (0.81, 2.25)	0.243	0.60 (0.32, 1.12)	0.107	**1.97 (1.12, 3.48)**	**0.019**
White	reference		reference		reference		reference		reference	
Household income										
$24,999 or less	**0.25 (0.20, 0.31)**	**<0.001**	**0.27 (0.22, 0.34)**	**<0.001**	**0.44 (0.35, 0.54)**	**<0.001**	**0.31 (0.24, 0.40)**	**<0.001**	**0.36 (0.28, 0.47)**	**<0.001**
$25,000 to $49,999	**0.30 (0.25, 0.37)**	**<0.001**	**0.32 (0.26, 0.39)**	**<0.001**	**0.47 (0.39, 0.57)**	**<0.001**	**0.50 (0.40, 0.62)**	**<0.001**	**0.43 (0.34, 0.54)**	**<0.001**
$50,000 to $74,999	**0.39 (0.32, 0.46)**	**<0.001**	**0.39 (0.32, 0.46)**	**<0.001**	**0.52 (0.43, 0.62)**	**<0.001**	**0.72 (0.60, 0.88)**	**0.001**	**0.56 (0.45, 0.71)**	**<0.001**
$75,000 to $99,999	**0.54 (0.46, 0.65)**	**<0.001**	**0.59 (0.49, 0.70)**	**<0.001**	**0.56 (0.47, 0.67)**	**<0.001**	**0.76 (0.63, 0.91)**	**0.004**	**0.75 (0.60, 0.93)**	**0.008**
$100,000 to $199,999	**0.70 (0.60, 0.81)**	**<0.001**	**0.77 (0.66, 0.90)**	**0.001**	**0.82 (0.71, 0.95)**	**0.009**	0.95 (0.82, 1.12)	0.561	0.93 (0.77, 1.11)	0.416
$200,000 or greater	reference		reference		reference		reference		reference	
Parent's highest education										
High school education or less	**0.65 (0.55, 0.76)**	**<0.001**	**0.57 (0.48, 0.68)**	**<0.001**	**0.64 (0.54, 0.76)**	**<0.001**	0.88 (0.72, 1.07)	0.192	**0.62 (0.51, 0.75)**	**<0.001**
College education or more	reference		reference		reference		reference		reference	

Bold indicates p<0.05. B=coefficient from linear regression model. Models represent the abbreviated output from the linear regression models with adjustment for age, sex, sexual orientation, race and ethnicity, household income, parent education, and site. ABCD propensity weights were applied to yield representative estimates based on the American Community Survey from the US Census.
